# *Fusobacterium nucleatum* and cancer

**DOI:** 10.1111/prd.12426

**Published:** 2022-03-04

**Authors:** Tamar Alon‐Maimon, Ofer Mandelboim, Gilad Bachrach

**Affiliations:** ^1^ The Institute of Dental Sciences The Hebrew University‐Hadassah School of Dental Medicine Jerusalem Israel; ^2^ The Concern Foundation Laboratories, Lautenberg Center for General and Tumor Immunology, Department of Immunology and Cancer Research, Institute for Medical Research Israel Canada (IMRIC), Faculty of Medicine The Hebrew University Medical School Jerusalem Israel

**Keywords:** *Fusobacterium nucleatum*, oncobacteria, oncobiont, pathobiont

## Abstract

Accumulating evidence demonstrates that the oral pathobiont *Fusobacterium nucleatum* is involved in the progression of an increasing number of tumors types. Thus far, the mechanisms underlying tumor exacerbation by *F. nucleatum* include the enhancement of proliferation, establishment of a tumor‐promoting immune environment, induction of chemoresistance, and the activation of immune checkpoints. This review focuses on the mechanisms that mediate tumor‐specific colonization by fusobacteria. Elucidating the mechanisms mediating fusobacterial tumor tropism and promotion might provide new insights for the development of novel approaches for tumor detection and treatment.

## 1 INFECTIVE AGENTS AND CANCER

In 1911, a causal role of microbes in cancer was first revealed by Peyton Rous who demonstrated that sarcoma can be induced in chickens by a virus.^1^ The link between a virus and human cancer was demonstrated 53 years later by Epstein, Achong and Barr as evidenced by the presence of Epstein–Barr virus in Burkitt lymphoma cells visualized by electron microscopy.^2^ This was followed with the association of hepatitis B and C viruses with liver cancer, papillomavirus with cervical cancer and herpesviruses with Kaposi sarcoma.^3^

In contrast to viruses, which play critical roles in cancer, bacteria were first considered as anti‐cancer agents (reviewed in reference^4^). In 1813, Vautier reported that patients with cancer who developed gas gangrene showed tumor regression.^5^ German physicians Busch and Fehleisen independently observed the regression of tumors in patients with cancer suffering from erysipelas infection. In 1868, Busch infected a cancer patient with erysipelas and noted tumor shrinkage. In 1882, Fehleisen repeated this treatment and identified *Streptococcus pyogenes* as the causative agent of erysipelas.^4^ Furthermore, in the United States in the early 1890s, a surgeon named William Coley pioneered the use of bacteria and their extracts (Coley's toxins) to evoke anti‐tumor immunity and successfully treat cancer patients.^6^ However, the high‐degree of success of newly developed radiation therapy led to a decline in the application of Coley's toxins as cancer treatment (reviewed in reference^7^). Bacterial‐based anticancer treatment reemerged in 1990, when the FDA approved the Bacillus Calmette–Guérin (BCG) vaccine, a live attenuated form of *Mycobacterium bovis* that is used against tuberculosis, for treating noninvasive bladder cancer.^8^, ^9^ Currently, BCG is the only anti‐cancer bacterial agent approved for routine clinical use.^4^ BCG, and fungal‐derived polysaccharide β‐glucan, can promote a sustained enhanced response of myeloid and natural killer (NK) cells to secondary infectious, inflammatory challenges, and tumors. This process of non‐specific memory of innate immune cells, facilitates the heightened response of these cells, as well as that of their progeny, to future challenges, and has been termed ‘‘trained innate immunity’’ or ‘‘innate immune memory’’.^10^, ^11^ Trained immunity is mediated via transcriptomic, epigenetic, and metabolic reprogramming.^11^ NK cells,^12^ and the induction trained immunity,^13^ are hypothesized to play important roles in BCG immunotherapy for noninvasive bladder cancer.^14^

The realization that *Helicobacter pylori* is a causative agent of gastric cancers in the 1990s indicated that bacteria are involved in tumor promotion.^15^, ^16^, ^17^, ^18^ Furthermore, mice that were genetically susceptible to cancer developed significantly fewer tumors under germ‐free conditions than those with conventional microbiota, thus supporting the pro‐tumorigenic roles of bacteria.^19^, ^20^ Studies employing advanced genomic sequencing and microbiome characterization methods indicate the association of bacterial species with specific cancers.^21^, ^22^ Multiple features of tumor, including proliferation, survival, progression, immunogenicity, sensitivity, and resistance to therapy, are affected by their interaction with the components of their microbial environment.^22^, ^23^ Although some bacterial species can promote cancer, those found to have reduced abundance in cancers might have cancer‐inhibitory actions or antagonistic interactions with tumor‐promoting bacteria.^24^, ^25^

Among the first bacteria suggested as potential cancer drivers are *Escherichia coli* strains that generate a mutagenic toxin called colibactin, which can induce single‐strand DNA breaks, and fragilysin‐expressing *Bacteroides fragilis*, which is genotoxic and can cleave the tumor suppressor protein E‐cadherin.^19^ *Streptococcus gallolyticus* (former *Streptococcus bovis*) bacteremia is an indicator of colorectal cancer since 1951^26^; however, the specific bacteria–cancer interaction is not understood. Overall, approximately 16% to 20% of cancer incidence can be linked to infectious agents.^27^, ^28^, ^29^ A recent report comprehensively characterized the microbiome of seven solid tumors.^21^

Cancer is among the comorbidities affected by periodontal pathobionts.^30^, ^31^, ^32^ *Fusobacterium nucleatum* the focus of the review, is an oral oncobiont mostly associated with the development of periodontitis. Highly abundant *F. nucleatum* has been detected in various types of cancer, including colorectal (CRC),^33^, ^34^ pancreatic,^35^, ^36^ esophageal,^37^, ^38^ and breast cancers,^39^, ^40^ and associated with shorter survival in patients with CRC, pancreatic, and esophageal cancers.^35^, ^37^, ^38^, ^41^, ^42^ Accumulating evidence indicating that *F. nucleatum* accelerates tumorigenesis^40^, ^43^, ^44^, ^45^, ^46^, ^47^, ^48^ and induces resistance to chemotherapy^49^, ^50^, ^51^, ^52^ may provide rational for the association of high amounts of *F. nucleatum* with poor disease outcome.

The mechanisms by which *F. nucleatum* accelerates tumor progression and metastasis and induces tumor‐chemoresistance have been thoroughly reviewed previously.^53^, ^54^, ^55^, ^56^, ^57^, ^58^, ^59^, ^60^, ^61^, ^62^, ^63^, ^64^, ^65^, ^66^, ^67^ This paper focuses on fusobacterial mechanisms that guide tumor‐specific colonization and protect tumors against anti‐tumor immunity.

## 2 *FUSOBACTERIUM NUCLEATUM* IN THE ORAL CAVITY

**FIGURE 1:**
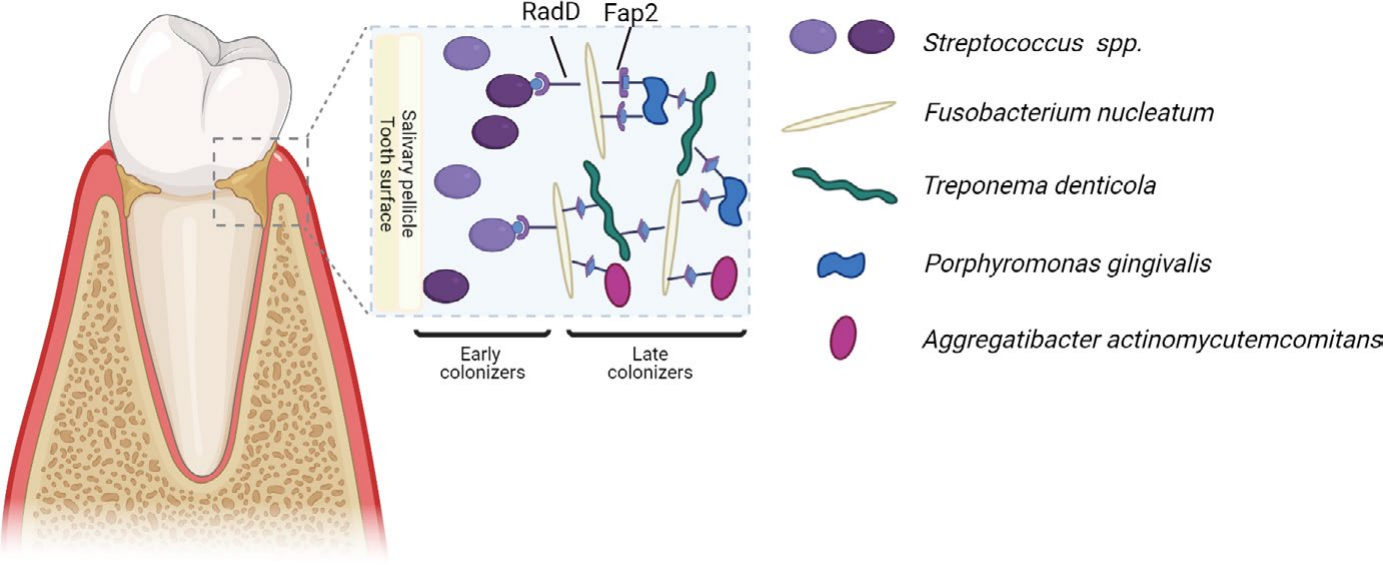
*F. nucleatum* acts as a bridging organism in dental plaques. A. Scanning electron microscopic image of a multispecies human oral biofilm. B. Schematic representation showing the ability of *F. nucleatum* to function as a “bridging” organism connecting the early colonizers, such as *Streptococcus* species via the RadD adhesin, and the largely anaerobic secondary colonizers, including *Porphyromonas gingivalis* via Fap2, *Treponema denticola,* and *Aggregatibacter actinomycetemcomitans*

*Fusobacterium nucleatum* is a gram‐negative, spindle‐shaped, non‐spore forming, oral anaerobe and is one of the most abundant gram‐negative species residing in the human oral cavity.^68^, ^69^ It is one of the pathobionts that outgrow during dysbiosis that precedes periodontal disease^68^, ^69^ and assist keystone species such as *Porphyromonas gingivalis* ^70^ in disrupting host–microbial homeostasis and inducing periodontitis.^71^, ^72^ It can be found on the dorsal surface of the tongue^73^, ^74^ and in multispecies biofilms at the gingival margin of the tooth, where it is hypothesized to play an important role in the development of the subgingival dental plaque. Owing to its abundant adhesion mechanisms, *F. nucleatum* can bind many oral bacterial species. Attachment between different oral colonizers is termed coaggregation or coadherence.^75^, ^76^, ^77^ By coaggregation with early oral colonizers capable of attaching to oral surfaces, such as *Streptococcus* species (via the RadD adhesin),^78^ and the largely anaerobic secondary colonizers that are associated with periodontal disease, including *Porphyromonas gingivalis* (via Fap2 as will be discussed below), *Treponema denticola,* and *Aggregatibacter actinomycetemcomitans,* and bridging them, *F. nucleatum* play a scuffle‐like, structurally supportive role in the oral biofilm that can resist washing by the saliva and gingival crevicular fluid. Multispecies bridging also facilitates multi‐species community existence, including communication, cross‐feeding, and metabolic interactions (Figure 1).^55^, ^75^, ^76^, ^79^

## 3 *FUSOBACTERIUM NUCLEATUM* IS OVERABUNDANT IN COLORECTAL CANCER

CRC is the second most common cause of cancer deaths in the United States^80^ and the fourth leading cause of cancer‐related deaths worldwide.^81^ The burden of CRC is rapidly increasing in developing countries as they adopt western lifestyles.^81^ In 2012, two studies employing applied computational approaches found increased fusobacteria (particularly *F. nucleatum*) DNA or RNA levels in colorectal cancer tissues compared to adjacent normal tissues.^33^, ^34^ This discovery was unexpected as fusobacteria are the core resident members of the human oral microbiome and infrequently found in the gut.^82^, ^83^ Live *F. nucleatum* directly isolated from biopsy samples^34^, ^84^, ^85^ and patient‐derived xenografts in mice^46^ confirmed these metagenomic results. Interestingly, the proportion of *F. nucleatum*–high colorectal cancers gradually increased from rectal cancers to the cecal cancers.^86^ Remarkably, a stronger association between *F. nucleatum* and CRC patients was found in Asiatic populations than in European and American populations (for a recent systematic review and meta‐analysis, please see references^42^, ^87^). In addition, *F. nucleatum* in CRC patients was frequently detected with other oral anaerobic species including *Peptostreptococcus* spp.^46^, ^88^ *Leptotrichia* and *Campylobacter*.^89^ Increasing evidence indicates that the presence of *F. nucleatum* in colon cancer is associated with resistance to chemotherapy, disease recurrence, and poor prognosis, which will be discussed in detail in section 9 below.

## 4 CRC‐ASSOCIATED *F. NUCLEATUM* ORIGINATES FROM THE ORAL MICROBIOTA

Although *F. nucleatum* is a common oral isolate, it is not abundantly found in the gut microbiota. Thus, fusobacteria detected in colon cancer samples are speculated to be of oral origin. To confirm this hypothesis, Komiya et al^90^ collected colon cancer specimens and matched saliva samples from 14 CRC patients and isolated *F. nucleatum* strains (*n* = 361) from the tumors of eight (57.1%) and the saliva of all 14 patients. Matching patterns of arbitrarily primed PCR products of tumor and oral isolates were found in six of eight (75%) patients thus suggesting that fusobacteria found in colon cancer tumors originated from the oral cavity. To further verify these results, Abed et al^85^ isolated the genomic DNA of *F. nucleatum* obtained from paired oral and adenocarcinoma samples from three patients. Genomic DNA was sequenced and compared with the available fusobacterial genomes deposited in the Sequence Read Archive (SRA) database. The results revealed the extremely close evolutionary relationship between each oral and matching tumor isolate, thereby supporting fusobacteria from the oral cavity may seed and become enriched in colorectal cancers.^85^ The frequent co‐occurrence of *F. nucleatum* in tumors with potential oral coaggregation partners, including *Peptostreptococcus* spp.^46^, ^88^ *Leptotrichia* and *Campylobacter* spp.,^89^ also substantiate the oral origin of colorectal cancer‐colonizing fusobacteria.

## 5 ORAL *F. NUCLEATUM* CAN TRANSLOCATE TO COLORECTAL TUMORS VIA THE HEMATOGENOUS ROUTE

Considering the oral origin of colon cancer‐associated fusobacteria, the route of their oral to tumor transmission remained to be resolved. Kostic et al^45^ demonstrated that oral fusobacteria can reach colon tumors by descending via the digestive tract.^45^ However, hematogenous translocation that can occur during frequent gingival bleeding^91^ is also possible. Such hematogenous transfer of oral fusobacteria to the placenta was previously observed, thus explaining its high occurrence in preterm births.^92^ (Reviewed in this volume by Y. W. Han).

Abed et al^85^ studied the preferred oral tumor route by employing two orthotropic mouse colon cancer models, namely MC38 in C57BL/6 mice and CT26 in BALB/C mice. They compared colon tumor colonization by *F. nucleatum* that was intravascularly injected via the tail vein or administered via oral gavage. Under the tested conditions, tumor colonization by the intravascularly injected fusobacteria is more efficient than that of the gavage‐inoculated ones in both mouse models.^85^ Intravenously injected fusobacteria were detected in mouse CT26 colon tumors at 2 h post‐delivery, and their levels remained stable at 6 h post‐infection. Fusobacterial proliferation in the tumor was observed at 24 h and 72 h post‐infection.^85^

The magnitude of bacteremia resulting after a dental procedure and routine daily activities is significantly lower (<10^4^ CFU/ml)^93^ than that tested in the experiments described above (1 × 10^7^–1 × 10^8^ *F. nucleatum* per mouse). However, when fusobacteria were inoculated in physiological doses in the orthotropic MC38 CRC model, tumor‐associated fusobacteria were also detected in mice inoculated with the more physiologic dose range (1 × 10^4^ *F. nucleatum* ^93^). Increased doses resulted in increased proportion of mice‐bearing tumors with intertumoral fusobacteria. In detail, fusobacteria were detected in the tumors of 45% of mice‐bearing tumors inoculated with 5 × 10^3^ to 1 × 10^4^ *F. nucleatum*; 60%, 5 × 10^4^ to 1 × 10^5^; and 100%, 5 × 10^6^ to 1 × 10^7^. Thus, lowering the fusobacterial inoculation dose did not suppress colon tumor colonization but rather reduced its efficiency. These results may explain the heterogeneity observed in fusobacterial occurrence in 3% to 56% of human colorectal cancer.^55^

The above results do not rule out that oral fusobacteria, which are constantly swallowed, may colonize colon tumors through the digestive tract. However, the hematogenous dissemination of oral fusobacteria to CRC is biologically conceivable as bloodstream travel circumvents the toxicity of low gastric pH and bile acids encountered upon descent to the gastrointestinal tract. Furthermore, bloodstream travel affords fusobacteria an escape from competition with the endogenous colonic microbiota.^85^

## 6 FAP2–GLYCANS INTERACTIONS GUIDE *F. NUCLEATUM* COLONIZATION IN COLORECTAL CANCER

Whether oral fusobacteria translocate to colon tumors via the blood circulation or descending through the digestive tract, mechanisms that home and localize fusobacteria to colorectal tumors must exist. Tumor‐induced conditions, including increased blood supply, blood vessel leakiness, hypoxia, and immunosuppressive microenvironment, are non‐specific factors that might contribute to a niche that promotes fusobacterial survival. However, these local environmental conditions are apparently not sufficient to enable the localization of other abundant oral anaerobic bacteria, such as *Porphyromonas gingivalis,* to colon cancers.^94^ Therefore, specific factors and mechanisms might be required for CRC colonization by fusobacteria. Current evidence suggests that tumor localization by *F. nucleatum* is dictated by glycan–lectin interactions.

D‐galactose‐β(1‐3)‐N‐acetyl‐D‐galactosamine (Gal‐GalNAc) or an unknown structural‐related sugar moiety is hypothesized as a tumor ligand for fusobacterial attachment. Gal‐GalNAc was found to be over‐displayed in sections of colorectal adenocarcinoma and has been suggested as a biomarker for colon cancer.^95^ GalNAc and Gal‐GalNAc are O‐GalNAc glycans and protein post‐translational modifications. In the biosynthesis of O‐GalNAc glycans, the first step involves the covalent linkage of N‐acetylgalactosamine (GalNAc) to selected Ser/Thr residues of the acceptor protein to yield GalNAcα1‐O‐Ser/Thr (also called the Tn‐antigen). A galactose (Gal) monosaccharide might then be linked to the GalNAcα1‐O‐Ser/Thr, consequently generating Galβ3GalNAcα1‐O‐Ser/Thr (Gal‐GalNAc‐O‐Ser/Thr), which is also called core 1 glycan, T‐antigen, or Thomsen–Friedenreich antigen.^96^ In normal cells, N‐acetylneuraminic acid, the predominant sialic acid in human and many mammalian cells, is frequently added to cap and mask the GalNAc and Gal‐GalNAc residues.^96^, ^97^ However, in many carcinomas (such as CRC), truncated O‐GalNAc glycans are formed, and sialic acid is not added to the exposed GalNAc and Gal‐GalNAc.^97^, ^98^ As a result, high levels of GalNAc (Tn antigen) and Gal‐GalNAc (T antigen) have been detected in colon cancer and additional human tumors including lung, breast and liver carcinoma.^96^, ^99^, ^100^ Such high levels of unmasked Tn‐ and T‐ antigens are associated with tumor invasion and metastasis.^99^

In the dental plaque, the coaggregation of *F. nucleatum* with many gram‐negative species can be inhibited by galactose and GalNAc indicating that *F. nucleatum* expresses a lectin (previously termed adhesin) that binds these sugar molecules present on the receptor of these coaggregation‐partner bacteria.^75^, ^101^ Transposon mutagenesis and mutant screening results identified the outer‐membrane Fap2 protein as the fusobacterial lectin that mediates GalNAc‐inhibited coaggregation.^102^ Interestingly, in previous studies, Fap2 was found to enable the ability of *F. nucleatum* to induce apoptosis in lymphocytes.^103^, ^104^ Therefore, it is plausible that Fap2 mediates the binding of *F. nucleatum* to lymphocytes, and enable additional fusobacterial factors to induce this apoptosis‐mediated immune evading mechanism.

As Gal‐GalNAc is over‐displayed by colon tumors, it has potential as an oncotarget for fusobacterial Fap2. In agreement with this, the attachment of *F. nucleatum* to colon cancer cell lines and colon cancer sections correlated with the amounts of Gal‐GalNAc detected on the target cells. In addition, its attachment was reduced upon O‐glycanase treatment and inhibited by soluble GalNAc in a dose‐dependent manner.^85^, ^94^ Fap2‐inactivated *F. nucleatum* mutants and clinical *F. nucleatum* isolates deficient in Fap2 hemagglutination activity exhibited impaired attachment to colon tumor cell lines and clinical specimens. More importantly, IV inoculated Fap2‐deficient *F. nucleatum* mutants were impaired in colonizing colon cancer mouse models.^85^, ^94^

## 7 GAL‐GALNAC IS OVER‐DISPLAYED IN MANY ADENOCARCINOMAS

Evidence suggests that oral *F. nucleatum* can hematogenously translocate to and specifically colonize colon cancer tumors^85^ via recognition and attachment to Gal‐GalNAc (or related sugars), which is highly displayed in colon cancer.^85^, ^94^ This indicates that *F. nucleatum* can reach other Gal‐GalNAc–displaying tumors through the same mechanism.

**FIGURE 2:**
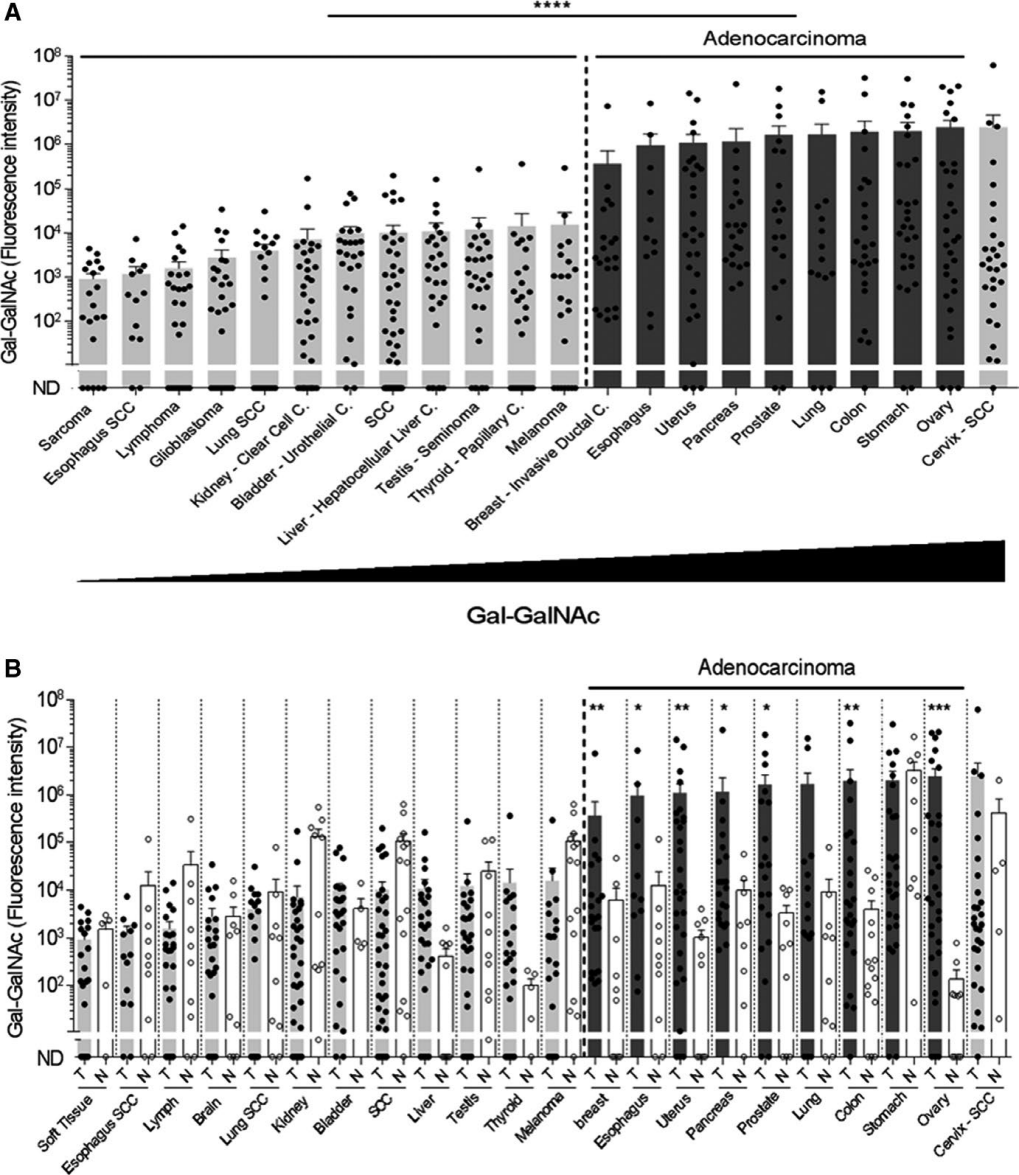
Gal‐GalNAc levels are increased in human adenocarcinomas. (A) Tumors are arranged according to increasing Gal‐GalNAc levels. Examined adenocarcinomas that displayed high levels of Gal‐GalNAc are marked with dark gray (right). (B) Gal‐GalNAc levels in the tumors (shaded dots) described in (A) were compared to those in matched normal tissue controls (hollow dots). Of the nine examined adenocarcinomas, seven showed significantly higher Gal‐GalNAc levels than the matched control tissues. The normal tissue controls for the esophagus, lung, and skin were used twice for the respective esophagus adenocarcinoma and esophagus squamous cell carcinoma (Esophagus SCC), the respective lung adenocarcinoma and lung SCC, and for the melanoma and SCC. Each symbol represents the fluorescent intensity of a sample from different patient. Data are presented as the mean ± SEM (*P < .05, **P < .01, ***P = .0001 analyzed by two‐tailed Mann‐Whitney test; ****P < .0001 analyzed by two‐tailed t‐test). This figure is from reference^105^

A screen for tumors that display high Gal‐GalNAc levels and might be targeted by fusobacteria was conducted, and Gal‐GalNAc levels of 20 different types of tumors were determined based on fluorescently labeled peanut agglutinin (PNA), a Gal‐GalNAc‐specific lectin.^105^ In agreement with previous reports,^99^ high Gal‐GalNAc levels were detected in 10 tumors types of epithelial tissues with glandular origin or/and characteristics (Figure 2A).^105^ Of which, nine were adenocarcinomas, namely that of the stomach, prostate, ovary, colon, uterus, pancreas, breast, lung, and esophagus. The remaining one was a squamous cell carcinoma of the cervix. In addition, Gal‐GalNAc levels were significantly higher in seven of these adenocarcinomas than in the matched normal control tissues (Figure 2B), whereas those in the stomach, lung, and cervix of the normal control samples were high and similar to those of their respective adenocarcinomas.^105^

Concurring with the speculation that fusobacteria can home‐in and accumulate in cancers that display high Gal‐GalNAc levels, fusobacterial DNA levels were reported to be overabundant in the pancreas,^35^, ^36^ esophagus,^37^ gastric,^106^, ^107^ cervical,^108^ and breast^39^ adenocarcinomas. Importantly, similar to its prevalence in colorectal cancer,^41^, ^109^ fusobacterial occurrence in pancreatic tumors was associated with shorter survival.^35^ High levels of *F. nucleatum* nucleic acids in esophageal cancer was also associated with shorter survival^37^ and poor response to neoadjuvant chemotherapy.^38^

Interestingly, high levels of Gal‐GalNAc are also found in the placenta,^110^, ^111^, ^112^ another extraoral niche, in which *F. nucleatum* is associated with pathology (Reviewed in this volume by Y. W. Han). Fap2‐inactivated mutants were deficient in placental colonization,^102^ suggesting that, Fap2–Gal‐GalNAc interaction might be involved in placental colonization by *F. nucleatum*, similar to tumor colonization.

## 8 BREAST CANCER COLONIZATION BY *F. NUCLEATUM*

**FIGURE 3:**
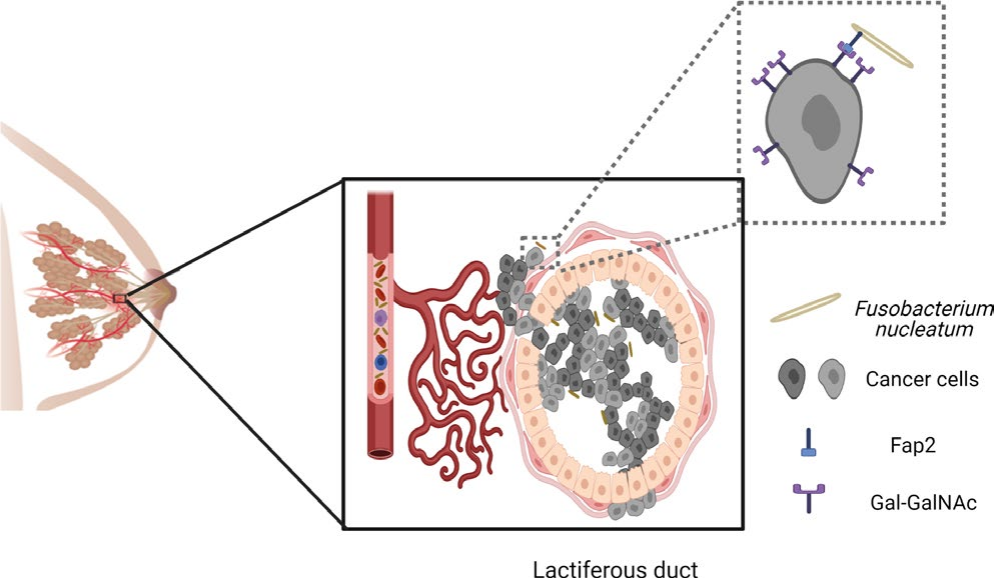
Breast cancer colonization by *F. nucleatum*. Schematic representation depicting the mechanism of the translocation of oral *F. nucleatum* to breast tumor via blood circulation. The bacterial lectin Fap2 enables the specific binding of *F. nucleatum* to cancerous cells that over‐display Gal‐GalNAc

*Fusobacterium nucleatum* is enriched in the breast cancer microbiome,^21^, ^39^, ^40^ which supports the hypothesis that fusobacteria can reach tumors via the circulatory system. A study focusing on breast cancer^40^ revealed that Gal‐GalNAc levels increase along with the progression of human breast cancer, similar to colon cancer ie, transition from adenoma to adenocarcinoma.^94^ The most dramatic rise in Gal‐GalNAc levels occurs in the transition from hyperplasia to atypical hyperplasia.^40^ Breast cancer, which develops in a sequence of events, begins with non‐neoplastic epithelial cells undergoing hyperplasia, atypical hyperplasia, carcinoma in situ, and eventually invasive adenocarcinoma. The conversion from benign hyperplasia to carcinoma in situ (the stage preceding invasive carcinoma) is speculated to occur at the transition from hyperplasia to atypical ductal hyperplasia.^113^ Importantly, the presence of *F. nucleatum* gDNA in breast cancer samples was correlated with high Gal‐GalNAc levels.^40^ In mouse models of breast cancer, when *fap*2‐expressing *F. nucleatum* ATCC 23726 was intravascularly inoculated, specific colonization of mammary tumors was observed (Figure 3). In contrast, *fap*2‐inactivated *F. nucleatum* mutants showed impaired tumor colonization.^40^ The inoculation of *F. nucleatum* into C57BL/6 mice orthotopically implanted with AT3 breast cancer cells resulted in the impaired accumulation of tumor‐infiltrating CD4+ and CD8+ T cells. Tumors obtained from *F. nucleatum*‐inoculated mice were significantly larger in volume than those from non‐inoculated ones. The progression of lung metastasis was also significantly enhanced in the *F. nucleatum* ‐ infected group. Fusobacterial‐induced breast tumor growth and metastatic progression in mice were revealed to be Fap2‐dependent and could be prevented by antibiotic treatment,^40^ suggesting that targeting *F. nucleatum* or Fap2 might be beneficial for the treatment of breast cancer. Although these results indicate the existence of *F. nucleatum* in human breast cancer, the possible role of fusobacteria in human breast cancer development and treatment outcome has not yet been investigated in the clinical setting.

## 9 TUMOR EXACERBATION BY *F. NUCLEATUM*

To date, *F. nucleatum* has been reported as overabundant in colon adenocarcinoma,^34^, ^45^ esophageal cancer,^37^ pancreatic cancer,^35^, ^36^ and breast cancer.^40^ Fusobacterial presence has been associated with poor prognosis in colon, rectal, pancreatic, and esophageal cancers^35^, ^37^, ^41^, ^109^, ^114^ and with treatment failure in colorectal and esophageal cancers.^38^, ^49^ In an animal model of colon and breast cancer, *F. nucleatum* accelerated tumor growth and metastatic progression.^40^, ^44^, ^45^, ^46^ Tumor acceleration by *F. nucleatum* involves the promotion of proliferation,^43^, ^44^ generation of a pro‐tumorigenic immune microenvironment,^45^ and the reduction in the number of tumor‐infiltrating lymphocytes (TILs).^40^, ^115^ *F. nucleatum* further inhibits the anti‐tumor activity of some TILs and NK cells that reach the tumor site by activating the human TIGIT checkpoint by utilizing a non‐lectin domain of the fusobacterial Fap2^116^ and the human CEACAM1 checkpoint via fusobacterial CbpF.^117^, ^118^, ^119^ In this section, we discuss these various mechanisms of tumor exacerbation induced by *F. nucleatum*.

### 9.1 *Fusobacterium nucleatum* enhances the proliferation of tumor cells

The FadA adhesin of *F. nucleatum* 12230 was shown to stimulate the proliferation of the human colon cancer cell lines HCT116, DLD1, SW480, and HT29 in a time‐dependent manner.^43^, ^44^ FadA interaction with E‐cadherin facilitated bacterial adhesion and invasion of E‐cadherin‐expressing cells via clathrin‐mediated endocytosis. Short incubation period of FadAc (the FadA active complex) with HCT116 cells impaired the tumor‐suppressing activity of E‐cadherin, resulting in the decreased phosphorylation of β‐catenin and subsequently increasing its stability and translocation into the nucleus. The nuclear translocation of β‐catenin activates the Wnt pathway and enhances the expression of NF‐κB and the oncogenes Myc and Cyclin D1. In agreement with these in vitro results, significant increases in *Fad*A, *Wnt*7b (a representative Wnt gene), and *NFkB*2 expression were detected in human cancerous colon tissues compared with normal ones.^44^ Annexin A1 was later revealed as a key component by which *F. nucleatum* exerts its stimulatory effect on cell proliferation. Downregulation of *ANXA1* (Annexin A1 gene) by siRNA effectively reduced *F. nucleatum* binding and invasion in a similar manner to the suppression of *CDH1*, which encodes E‐cadherin.^43^ These findings are supported by an independent study demonstrating that recombinant FadA promotes the proliferation of SW480 colon cancer cells in a dose‐ and time‐dependent manner.^48^

*Fusobacterium nucleatum* can also enhance the proliferation and invasion of colon cancer cells by upregulating microRNA 21 (miR21).^47^ A microRNA screening of four human colorectal cancer cell lines, including HCT116, HT29, LoVo, and SW480, revealed that miR21 is the most upregulated miRNA upon incubation with *F. nucleatum*. *F. nucleatum* increases the expression of miR21 by activating the MYD88‐dependent Toll‐like receptor 4 signaling pathway, thus upregulating the nuclear factor‐κB (NF‐κB) signaling pathway. MiR21 decreases the levels of RAS GTPase encoded by *RASA1*, thus activating the RAS‐mitogen‐activated protein kinase (MAPK) cascade.^120^, ^121^ Consistently, the inhibition of miR21 suppressed cell proliferation and invasion. Analysis of 90 human‐matched CRC and normal tissues revealed that *F. nucleatum* DNA and miR21 transcripts were more abundant in cancer tissues than the control and that their levels were significantly higher in more advanced tumors. Importantly, high levels of *F. nucleatum* DNA and miR21 in tumors correlated with shorter survival.^47^

### 9.2 *Fusobacterium nucleatum* promotes chemoresistance in CRC

Resistance to chemotherapy is a major cause of tumor recurrence and poor prognosis in patients with CRC. As the abundance of *F. nucleatum* has been reported in the CRC tissues of post‐chemotherapy recurrence patients compared to non‐recurrence patients, studies have explored whether fusobacteria are involved in chemoresistance.^49^

Oxaliplatin and 5‐fluorouracil (5‐FU) are widely used for CRC treatment. 5‐FU inhibits the activity of thymidylate synthase during DNA replication,^122^ and oxaliplatin covalently binds DNA and forms platinum‐DNA adducts, resulting in cell‐cycle arrest at G2 phase.^123^ Infecting HCT116 and HT29 human colon cancer cell lines with *F. nucleatum* induced the expression of the LC3‐II marker of autophagosomes,^124^ suggesting that fusobacteria might induce colorectal cancer chemotherapeutic response. Moreover, the cytotoxicity of oxaliplatin or 5‐FU treatment on *F. nucleatum‐*infected colon cancer cells was significantly reduced. Meanwhile, the addition of chloroquine (CQ), an autophagy lysosomal inhibitor, restored drug cytotoxicity. Following *F. nucleatum* exposure, the expression of miR‐18a and miR‐4802 was the most significantly downregulated among miRNAs in the tumor cells, and their levels inversely correlated with those of the autophagy elements ULK1 and ATG7. The *F. nucleatum*‐induced reduction in miR‐18a and miR‐4802 levels was dependent on the TLR4/MYD88 signaling pathway. The proposed mechanism speculates that exposure of cancer cells to *F. nucleatum* activates the TLR4 and MYD88 signaling pathways to downregulate the expression of miR‐18a and miR‐4802, thus inducing a switch from apoptosis to autophagy and drug resistance.^49^, ^125^

Additional mechanisms by which *F. nucleatum* regulate apoptosis to induce alterations in chemosensitivity to 5‐FU have also been described. For example, *F. nucleatum* infection has been reported to upregulate BIRC3 via the TLR4/NF‐kB signaling in HCT116 and HT29 cells. BIRC3, a member of the inhibitor of apoptosis protein (IAP) family, can suppress apoptosis by directly inhibiting the caspase cascade.^126^ A SMAC mimetic, a small molecule antagonist of BIRC3, gradually diminished chemoresistance induced by *F. nucleatum*. In human CRC tissues, high levels of *F. nucleatum* correlated with high levels of BIRC3. Moreover, high levels of *F. nucleatum,* TLR4, and BIRC3 were more likely to be detected in CRC patients with recurrence than in those without.^50^ In another study, the incubation of HCT116 and HT29 cells with *F. nucleatum* significantly upregulated the expression of anoctamin‐1 (*ANO1*), which encodes a human chloride channel protein. *ANO1* is located on chromosome 11q13, which is frequently amplified in different types of human carcinomas including head and neck squamous cell carcinoma, bladder cancer and breast cancer.^127^, ^128^ *F. nucleatum*‐infected cells treated with oxaliplatin or 5‐FU showed significantly lower levels of apoptosis. Silencing *ANO1* blocked the protective effect of *F. nucleatum* and increased apoptosis, whereas its overexpression further increased *F. nucleatum*‐induced chemoresistance.^51^

*Fusobacterium nucleatum*‐induced autophagy‐mediated chemoresistance has also been described in esophageal squamous cell carcinoma (ESCC). ESCC patients with high levels of *F. nucleatum* exhibited higher resistance to chemotherapeutic treatment than patients with lower levels of *F. nucleatum*. LC3, an autophagy marker, was predominantly detected in *F. nucleatum*‐treated ESCC cells compared to the control. Furthermore, in TE8 and TE10 human ESCC cells the expression of *ATG7*, an essential factor for the induction of autophagy, was significantly increased after incubation with *F. nucleatum*. Upon treatment with docetaxel, cisplatin (CDDP), or 5‐FU, which are key chemotherapeutic agents for ESCC,^129^, ^130^ *F. nucleatum*‐infected TE8 and TE10 cells showed significantly higher growth rate than the non‐infected control cells. CQ addition to the infected cells decreased cell growth. In agreement with these in vitro results, a positive correlation between *F. nucleatum* and the levels of the autophagy markers ATG7 and LC3 was observed in human ESCC tissues.^52^

### 9.3 *Fusobacterium nucleatum* establishes a tumor‐permissive immune microenvironment

Immune cells and their effectors play critical role in tumor control and progression. The ability of *F. nucleatum* to manipulate the tumor immune microenvironment was first demonstrated in a C57BL/6 *Apc^Min/+^* mouse model of intestinal tumorigenesis.^45^ Adenomatous polyposis coli (*Apc*) is a tumor suppressor gene and C57BL/6 *Apc* ^Min/+^ mice spontaneously develop intestinal cancers. Repeated oral inoculations with *F. nucleatum* but not with *Streptococcus* *sanguinis* (control), increased tumor multiplicity in these mice. Tumor promotion by fusobacteria involved the selective recruitment of tumor‐infiltrating myeloid cells, which can promote tumor progression. This was concluded due to the elevated number of infiltrating myeloid‐derived cells, which suppress CD4+ T cells, in the fusobacterial‐infected mice.^45^ In addition, the expansion of tumor‐associated neutrophils (TANs), which promote tumor progression and angiogenesis and impair antitumor immunity, and tumor‐associated macrophages (TAMs; both TAMs and M2‐like TAMs), which suppress T cell activity, was elevated in *F. nucleatum*‐infected mice‐bearing tumors compared to the controls. Analysis of the transcriptome sequencing data revealed that tumors from *Apc* ^Min/+^ mice exposed to *F. nucleatum* exhibited a proinflammatory expression signature that is shared with human fusobacteria‐positive colorectal carcinomas. Transcriptomic analysis of human colon tumors with high fusobacterial RNA levels revealed the *Fusobacterium*‐induced genes, PTGS2 (COX‐2), IL1β, IL6, IL8, and TNF (TNF‐α), indicating an NF‐κB‐driven proinflammatory response associated with colorectal carcinogenesis.^45^

### 9.4 *Fusobacterium nucleatum* inhibits the recruitment of anti‐cancer tumor‐infiltrating T cells

Accumulated evidence indicates that tumor‐colonized *F. nucleatum* can also interfere with the recruitment of TILs. In colorectal carcinoma tissues, the abundance of *F. nucleatum* was inversely correlated with T‐cell density.^115^, ^131^, ^132^ In post‐neoadjuvant locally treated advanced rectal cancer, fusobacterial persistence was associated with a lack of CD8^+^ T cells.^109^

In an AT3 orthotropic mouse model of breast cancer, *F. nucleatum* accelerated cancer progression by inhibiting the recruitment of TILs. C57BL/6 mice implanted with AT3 cells and IV‐inoculated with *F. nucleatum* showed significantly larger tumors and more lung metastasis than noninfected mice. Metronidazole treatment diminished the pro‐tumorigenic effects of the bacteria. Bacterial‐induced tumor enlargement was attributed to the inhibition of T cell recruitment into the tumor site as evidenced by the fewer number of CD4+ and CD8+ T cells detected in the tumors of *F. nucleatum*‐infected mice. Similarly, fusobacteria did not induce tumor enlargement when AT3 cells were implanted in SCID beige mice lacking T, B, and NK cells.^40^ Thus, these findings indicate that in immunocompetent C57BL/6 mice, the growth of AT3 breast tumor is restricted by NK, B, or T cells. However, in the presence of *F. nucleatum,* T cell levels were reduced, resulting in increased tumor growth. The reduction in the number of immune cells may involve apoptosis. Apoptosis was induced by *F. nucleatum* in lymphocytes via Fap2.^103^ Importantly, the immunomodulated pro‐tumorigenic effect of *F. nucleatum* is expected to be more significant in humans because the activity of NK and some T cells in tumors can be further weakened by the inhibitory interactions between Fap2 and TIGIT^116^ and between CbpF and CEACAM^117^ checkpoints (as discussed below).

### 9.5 *Fusobacterium nucleatum* activates immune checkpoints

While the presence of *F. nucleatum* in human colorectal cancer^115^, ^131^, ^132^ and in a mouse model of breast cancer^40^ has been associated with decreased number of TILs in the tumor site, the effect of fusobacteria on the recruitment of NK cells to tumors has not yet been reported. Remarkably, the tumor‐killing effect of NK cells on various tumor cell lines was inhibited by the presence of various *F. nucleatum* strains.^116^ To prevent autoimmune reactions and the killing of normal cells, the activity of immune cells can be negatively regulated by a large repertoire of inhibitory receptors, one of which includes TIGIT, an inhibitory receptor expressed by many immune cells, including NK cells. Tumor‐attached *F. nucleatum* inhibited immune cell activity via the interaction between the fusobacterial Fap2 protein and the human TIGIT inhibitory receptor.^116^ More recently, the suppression of immune cell anti‐tumor activity by *F. nucleatum* through the activation of an additional immune cell suppressing receptor CEAMAM1, was reported.^117^, ^119^ Thus, in addition to reducing the number of immune cells infiltrating the fusobacterial‐colonized tumor microenvironment, fusobacteria can further protect tumors by activating checkpoints to suppress immune‐cell anti‐tumor activity.

### 9.6 *Fusobacterium nucleatum* promotes metastasis

*Fusobacterium nucleatum* has been detected in CRC metastases to the liver and lymph nodes^33^, ^34^, ^46^, ^94^ and is associated with increased number of liver metastases in colorectal cancer.^46^, ^133^ In a mouse model of breast cancer, *F. nucleatum* promoted lung metastasis.^40^ The presence of *F. nucleatum* was also shown to promote the successful establishment of CRC patient‐derived xenografts in mice.^46^ The proposed mechanism by which *F. nucleatum* promotes metastasis involves the induction of proinflammatory cytokines that stimulate tumor cell migration and invasion. *F. nucleatum*‐infected CRC cells secrete cytokines IL‐8 and CXCL1, which promote the invasive motility of infected and non‐infected cells.^134^ Upon incubation with *F. nucleatum,* human and mouse breast cancer cells also induced the overexpression and increased secretion of the matrix metalloproteinase 9 (MMP‐9).^40^ Proteases of the MMP family play vital roles in many biological processes that involve matrix remodeling. In particular, MMP‐9 activity has been related to cancer pathology, including invasion, angiogenesis, and metastasis.^135^ Therefore, in addition to immune modulation, which is the putative major mechanism of *F. nucleatum* action in AT3 breast cancer model in C57BL/6 mice, the induction of MMP might be another mechanism by which *F. nucleatum* accelerates breast tumor progression.

Generally, metastasis is responsible for more than 90% of cancer‐associated mortality and is the main cause of breast cancer‐related deaths. Patients with localized breast cancer have a 5‐year survival rate of 98%, which dramatically decreases to 26% in patients with metastatic breast cancer.^136^ More studies are required to completely understand the pro‐metastasis mechanisms of *F. nucleatum*.

## 10 *FUSOBACTERIUM NUCLEATUM* AS A POTENTIAL DIAGNOSTIC BIOMARKER

Microbiome‐based oncology diagnostics are promising novel approaches for tumor detection. A recent report demonstrated the potential of plasma‐derived, cell‐free microbial nucleic acids for tumor screening. Good discrimination was achieved between samples from donors with tumors and those from healthy ones and among 32 different cancer types.^137^ Therefore, the overabundance of *F. nucleatum* in tumors can be utilized as a strategy for tumor detection. Although a number of approaches have been explored, adequate screening capabilities have not yet been achieved.

### 10.1 Stool screening for CRC detection

The early detection of cancers is important to reduce CRC mortality.^138^ Fecal occult blood testing (FOBT) is a common non‐invasive cost‐efficient method to screen for CRC^138^; However, FOBT has moderate sensitivity.^138^, ^139^, ^140^

Almost a decade ago, *F. nucleatum* was reported to be enriched in stool samples from colorectal adenoma and carcinoma patients compared to healthy subjects.^45^ Many reports have since corroborated this finding, particularly those involving Asian cohorts.^141^ A recent review and meta‐analysis demonstrated the potential of a fecal *F. nucleatum* ‐based test for detecting colorectal cancer; however, additional clinical trials should be performed to verify this.^141^

The combination of fecal quantification of *F. nucleatum* and FOBT was shown to increase the specificity and sensitivity of the latter,^142^, ^143^ indicating the applicability of this combination method as a large population‐based screening strategy employing large non‐invasive samples for colorectal cancer. To date, the quantification of *F. nucleatum* has been performed using quantitative PCR.^141^ It is expected that future developments of novel antibody‐ or enzymatic‐based assays might enable the combination of FOBT with fecal fusobacterial testing (FFT) in a single test.

### 10.2 Tumor detection based on antibody responses

Immune assays based on the serum, salivary, or fecal anti‐ *F. nucleatum* antibodies may also offer new opportunities for CRC screening. Thus far, serum anti‐ *F. nucleatum* antibodies could not discriminate between CRC patients and the controls with sufficient specificity and sensitivity.^144^, ^145^, ^146^ One study used multiplex serology assay to simultaneously measure antibody responses to 11 *F. nucleatum* recombinant antigens in prediagnostic serum samples from colorectal cancer patients and matched controls (n = 485 each). However, colorectal cancer risk was not significantly associated with antibody response to each *F. nucleatum* protein or combined positivity to any of the 11 proteins.^145^ In a subsequent study, ELISA‐based testing found that the levels of *F. nucleatum* IgA and IgG antibodies in the CRC group were higher than those in the healthy controls. However, the discriminative ability of the ELISA test was not adequate for diagnosis.^146^ Notably, plasma anti‐ *F. nucleatum* IgG level and salivary IgA level against *F. nucleatum* and specifically against Fap2, has been recently reported to be associated with pancreatic malignancy.^147^ However, the diagnostic potential of these findings should be confirmed by future studies.

## 11 ANTI‐TUMOR THERAPEUTIC STRATEGIES EMPLOYING *F. NUCLEATUM*

### 11.1 Elimination of tumor‐colonized *F. nucleatum*

As mentioned above (section 9), high fusobacterium load in tumors has been associated with poor disease outcomes in humans.^35^, ^37^, ^38^, ^41^, ^42^, ^49^, ^51^, ^52^ In animal models, systemic antibiotic treatment eliminated tumor‐colonized fusobacteria and subsequently suppressed fusobacterial‐induced tumor exacerbation, suggesting the effectivity of antibiotic treatment for cancer patients.^40^, ^46^ Unfortunately, in some cases, antibiotics might interfere with anti‐tumor treatment. Gut microbiota can influence anti‐tumor chemotherapy,^148^, ^149^ immunotherapy,^150^, ^151^, ^152^, ^153^, ^154^, ^155^, ^156^ radiotherapy,^157^ and allogeneic bone marrow transplantation^158^ via various proposed mechanisms.^23^ Fecal transplantation to restore the gut microbiota following antibiotic treatment might address this issue, especially if in the future, fecal transplant will be considered to aid anti‐cancer (chemotherapeutic, immunotherapeutic) treatments.^25^ Bacteriophages are viruses that prey and replicate in bacteria. The use of bacteriophages for targeting specific oncobacteria, including tumor‐colonized *F. nucleatum,* has been recently suggested.^25^ Importantly, a fusobacteria bacteriophage with a potential to eradicate tumor‐colonized *F. nucleatum* has been recently identified.^159^

### 11.2 Tumor targeting strategies using *F. nucleatum* and Fap2

Due to their specific homing to glycan‐overdisplaying tumors, *F. nucleatum* or Fap2 could potentially be used as a platform for targeting tumors and metastases that display high levels of Gal‐GalNAc (or related sugars). Recent advances in the genetic manipulation of *F. nucleatum* ^134^, ^160^ have facilitated the ability to weaken fusobacterial tumor‐enhancing actions in the future by for example mutating FadA and/or nullifying TIGIT and CEACAM1 activation. Such enfeebled strains can then be engineered to express anti‐cancer payloads. Possible anti‐tumor agents might include antigens that induce trained innate immunity, or antigens that induce innate and adaptive anti‐tumor immune responses, and/or enzymes that locally convert a nontoxic prodrug to a cytolytic drug. Such strategies are currently being tested with several tumor‐colonizing bacteria including *Salmonella* and *Listeria* (reviewed in^5^).

Importantly, live bacteria are currently used for cancer treatment.^161^ In case of adverse effects, this treatment can be terminated using antibiotics. For over three decades, the intravesical administration of live bacillus Calmette–Guérin, a vaccine against tuberculosis, has been used to treat bladder cancer.^161^ Anecdotally, bladder cancer patients treated with BCG have significantly reduced risk of Alzheimer's disease and Parkinson's disease compared to those not treated with BCG. The beneficial effect of BCG on neurodegenerative diseases has been attributed to the possible activation of long‐term nonspecific immune effects.^162^

A more advanced version of this tumor‐targeting approach might be targeting tumor‐colonized fusobacteria with bacteriophages engineered to express anti‐cancer payloads such as described above. A phage‐guided encapsulation of the anti‐tumor drug irinotecan dextran nanoparticles has been recently proposed to promote the growth of tumor‐suppressing *Clostridium butyricum*. The engineered nanocapsules were covalently bound to a phage that target the tumor‐colonized fusobacteria. The capacity of the phage‐guided nanoparticles to control tumor growth was then demonstrated in two mouse models of colon cancer.^163^

Similar to *F. nucleatum, Plasmodium falciparum,* the causal agent of malaria, is found in both the placenta and tumors. In the analogues of Fap2, VAR2CSA is the malaria protein speculated to be responsible for the accumulation of malaria‐infected erythrocytes to the placenta and tumors. During pregnancy‐associated malaria, malarial parasites express VAR2CSA proteins on the surface of infected erythrocytes. VAR2CSA enables the specific anchoring of the infected erythrocytes to the syncytiotrophoblast in the placenta by binding to chondroitin sulfate. Similar to the Fap2 oncofetal ligand Gal‐GalNAc, chondroitin sulfate is an oncofetal antigen shared between placental trophoblasts and cancer cells.^164^, ^165^

Recombinant VAR2CSA (rVAR2) coupled to magnetic beads can capture circulating tumor cells in a blood sample, thus serving as a potential tool for novel cancer diagnostics.^166^ The conjugation of a toxin to rVAR2 can also direct anti‐tumor therapeutics.^165^, ^167^ The parallel roles played by VAR2CSA–chondroitin sulfate and Fap2–Gal‐GalNAc interactions are interesting and require further investigation. The complementary utilization of Fap2 for tumor detection and treatment should also be explored.

## 12 CONCLUDING REMARKS

The terms alpha‐bugs^168^ also referred to as bacterial drivers^19^ have been proposed to describe certain members of the microbiome that possess direct pro‐oncogenic features or the ability to shift the local bacterial community to one that promotes mucosal immune responses and epithelial cell changes, consequently resulting in the development of colorectal cancer. Alpha‐bugs have been also suggested to enhance carcinogenesis by selectively “crowding out” cancer‐protective microbial species.^168^ “Classical” bacterial drivers possess virulence factors that might initiate cancer formation. These factors include the colibactin genotoxin of several *E. coli* strains that can induce single‐strand DNA breaks^169^ and the *B. fragilis* toxin fragilysin (BFT). BFT, a metalloproteinase, is genotoxic to colonic epithelial cells, upregulates spermine oxidase, a polyamine catabolic enzyme that contributes to increased production of reactive oxygen species and DNA damage.^170^ Fragilysin also promotes the proliferation of intestinal epithelial cells in a mechanism involving cleavage of the tumor suppressor protein E‐cadherin.^171^, ^172^

Currently, *H. pylori* is the only bacterium that is classified as a direct carcinogen. Epidemiological evidence and experimental data indicate that prevalence of *H. pylori* is associated with the development of gastric adenocarcinoma and gastric mucosa‐associated lymphoid tissue (MALT) lymphoma.^173^ *H. pylori* in the stomach mucosa is crucial in the chronic inflammatory process, which leads to gastric cancer development.^173^ Thus, the cytotoxin‐associated gene A (CagA) protein of *H. pylori*, which is delivered to gastric epithelial cells via bacterial type IV‐secretion, is an oncoprotein that can induce malignant neoplasms in mammals.^174^, ^175^

**FIGURE 4:**
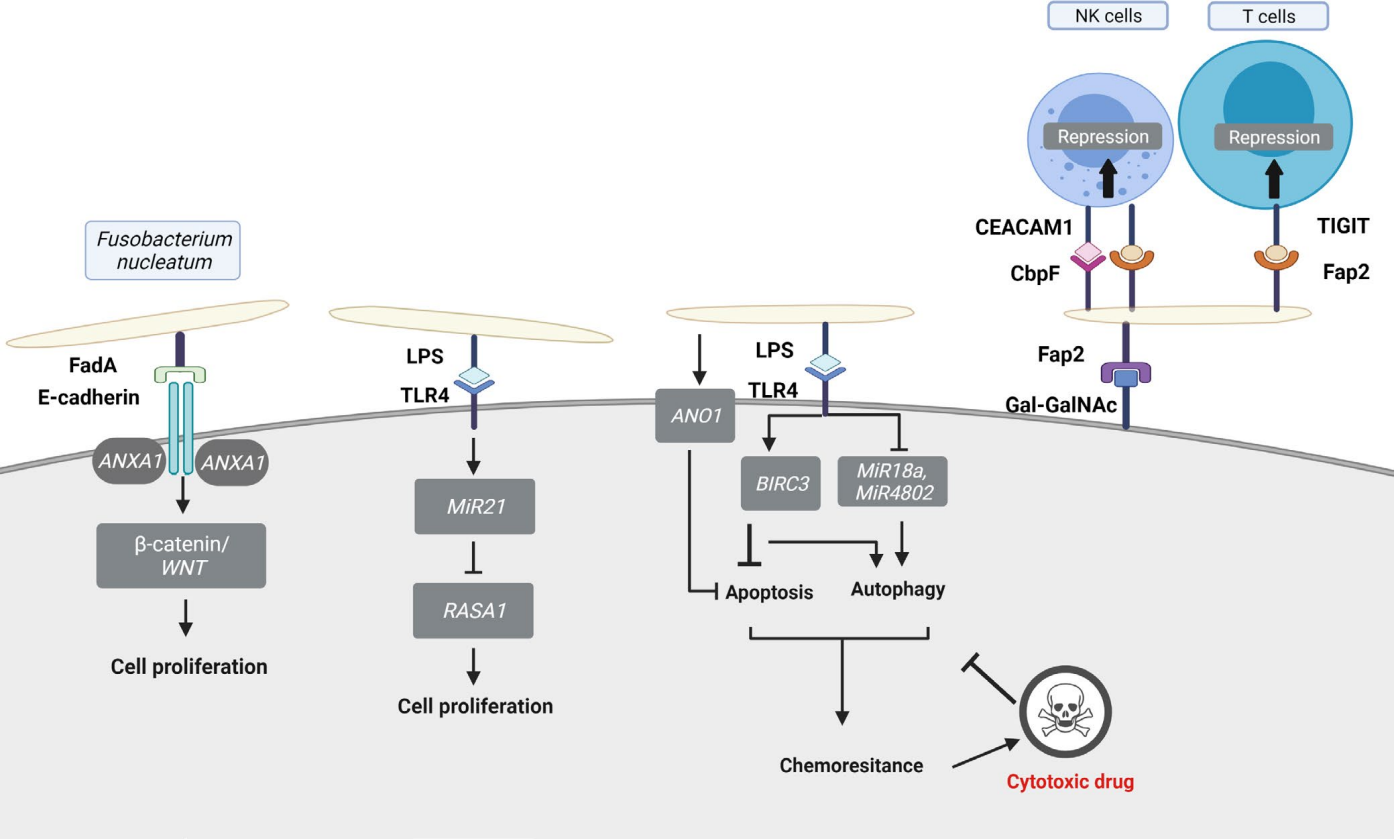
Various mechanisms utilized by *F. nucleatum* to accelerate tumor progression. The fusobacterial Fap2 domain binds tumor‐displayed Gal‐GalNAc to enable tumor colonization.^40^, ^94^ Tumor acceleration is then mediated with the following mechanisms

Unlike the cancer drivers mentioned above, based on the current evidence, *F. nucleatum* is a “passenger”^19^ bacteria that colonizes an already formed tumor and accelerates its progression through manipulation of β‐catenin signaling,^43^, ^44^ host cytokine production (IL‐8 and CXCL1),^134^ anti‐tumor immunity, and chemoresistance. These mechanisms are illustrated in Figure 4.

Occurrence of *F. nucleatum* is found to be associated with poor disease outcome in an increasing number of tumor types suggesting that targeting intratumor fusobacteria will improve prognosis.

High Gal‐GalNAc level is found in all tumor‐types colonized by fusobacteria indicating that it is an oncoantigen that plays a role in the specificity of tumor colonization by fusobacteria by serving as a ligand to Fap2. It is therefore tempting to assume that fusobacterial overabundance will be found in all Gal‐GalNAc overdisplaying tumors. Due to the tumor specificity, fusobacteria and Fap2 hold potential for use for tumor screening and treatment.

The fusobacterial adhesin FadA binds to E‐cadherin and activates the β‐catenin/WNT signaling pathway, thus promoting cell proliferation.^43^

The bacterial endotoxin lipopolysaccharide (LPS) activates the Toll‐like receptor 4 (TLR4) to trigger the upregulation of miR21. This decreases the levels of RAS GTPase *RASA1* and activates the RAS–mitogen‐activated protein kinase (MAPK) cascade to enhance cell proliferation.^120^, ^121^

*Fusobacterium nucleatum* LPS interactions with TLR4 can also upregulate BIRC3, which inhibits apoptosis by directly inhibiting the caspase cascade, thereby increasing cell resistance against cytotoxic drugs.^50^ In addition, LPS/TLR4 interactions downregulate the expression of miR18a and miR4802, which is associated with that of autophagy elements ULK1 and ATG7, resulting in increased autophagy and subsequently enhancing cell resistance to therapy.^49^, ^125^

Lastly, *F. nucleatum* inhibits apoptosis by upregulating the expression anoctamin‐1 (*ANO1*) in a TLR4‐dependent manner to contribute to chemoresistance.^51^

The non‐lectin domain of Fap2 inhibits the anti‐tumor activity of TILs and NK cells at the tumor site by activating the human TIGIT checkpoint.^116^

Fusobacterial CbpF further suppresses the anti‐tumor activity of TILs and NK cells by activating the human CEACAM1 checkpoint.^117^, ^118^
